# Cognitive behavioral therapy for pediatric obsessive-compulsive disorder delivered via internet videoconferencing: a manualized sensor-assisted feasibility approach

**DOI:** 10.1186/s13034-024-00844-7

**Published:** 2024-12-04

**Authors:** Carolin S. Klein, Annika K. Alt, Anja Pascher, Jan Kühnhausen, Lennart Seizer, Winfried Ilg, Annika Thierfelder, Jonas Primbs, Michael Menth, Gottfried M. Barth, Caterina Gawrilow, Annette Conzelmann, Tobias J. Renner, Karsten Hollmann

**Affiliations:** 1 Department of Child and Adolescent Psychiatry, Psychosomatics and Psychotherapy, University Hospital of Psychiatry and Psychotherapy, Osianderstr. 14-16, 72076 Tübingen, Germany; 2 DZPG (German Center for Mental Health), Partner site Tübingen, Tübingen, Germany; 3 Hertie Institute for Clinical Brain Research, Section for Computational Sensomotorics, University of Tübingen, Tübingen, Germany; 4 Department of Computer Science, Communication Networks, University of Tübingen, Tübingen, Germany; 5 Department of Psychology, University of Tübingen, Tübingen, Germany; 6 Department of Psychology (Clinical Psychology II), PFH– Private University of Applied Sciences, Göttingen, Germany

**Keywords:** Obsessive-compulsive disorder, Manualized treatment, Sensor technology, Internet-based cognitive behavioral therapy, Children and adolescents

## Abstract

**Background:**

Between 1 and 4% of children and adolescents suffer from obsessive-compulsive disorder (OCD) worldwide, but the majority of these young people do not have access to cognitive behavioral therapy (CBT) as a first-line treatment. CBT delivered via online videoconferencing (vCBT) offers a new way to provide young people with therapy, especially in the home environment where symptoms usually occur.

**Methods:**

In this study, we investigated the feasibility of a newly revised vCBT manual, symptom change during treatment, and effects on family life and social functioning. 20 patients with OCD, aged 12–18 years, were treated during 14 weekly sessions while using a multimodal sensor system that assessed their physiological and behavioral responses during therapy. Treatment was delivered in real time via an online videoconferencing platform. Measurements of feasibility, acceptance, and implementation were evaluated descriptively, and clinical measures were assessed with *t* tests.

**Results:**

The primary results showed that patients and parents perceived the manual-based vCBT as feasible and easy to understand. According to the therapists’ ratings, all treatment modules and the content could be carried out in accordance with the manual. As a secondary outcome, OCD symptoms improved significantly during treatment (*p* <.001, *d* = 1.87), revealed by an average decrease of more than half in the Children’s Yale-Brown Obsessive-Compulsive Scale (CY-BOCS) score. As the psychotherapy could be implemented directly in the patients’ home environment, low barriers to participation were reported, and the majority of participants reported improvements in family life after treatment.

**Conclusions:**

In summary, the results of this feasibility study indicated a successful application of manual-based psychotherapy delivered via videoconferencing for pediatric OCD supported by a sensor system. This method should be further investigated in future randomized controlled trials with larger patient samples.

*Clinical trial registration*: [www.ClinicalTrials.gov], identifier [NCT05291611], first submission: 2021-12-10.

## Background

An increasing number of children and adolescents around the world are affected by mental disorders [1–3]. If left untreated, mental illnesses can become chronic and have a significant impact on young people’s quality of life and social functioning, which may have far-reaching consequences for individuals and society as a whole [4, 5]. However, there are several barriers to treatment participation, such as long waiting lists, little knowledge about psychological issues, shame, logistical problems for the family, and a lack of trained psychotherapists [6–8]. Thus, new approaches to psychotherapeutic treatment are needed to help young people set a course for a healthier future, and online approaches may be one option, especially because children in particular have grown up as digital natives and are thereby capable of finding their way around the online world [9]. In particular, CBT delivered via videoconferencing (vCBT), where treatment is carried out in real time via an online videoconferencing platform, seems to have clinical outcomes that are similar to those of traditional face-to-face psychotherapy [10].

Moreover, some psychiatric disorders are even most effectively treated online at home because the home is where symptoms occur naturally, and therapeutic interventions can be applied directly in the triggering situation [11]. An example of a mental health condition for which treatment via videoconferencing is promising is obsessive-compulsive disorder (OCD), which is characterized by unpleasant, recurring thoughts that impose themselves against the will of those affected and repetitive behaviors, resulting in severe psychological stress and substantial functional impairment [12–14]. OCD in childhood and adolescence occurs with a prevalence of 1% in prepubertal children up to 4% in adolescents [15], with an average age of onset between 8 and 11 years [16]. Cognitive behavioral therapy (CBT) has proven to be the first-choice treatment method for OCD [17–19]. As part of CBT, exposure and response prevention (ERP) in particular is considered a core element in the treatment of OCD [17, 20], where patients confront themselves with the triggering stimuli without performing the compulsive acts until the tension has significantly decreased. On the basis of our clinical impression from our specialized outpatient clinic as well as exchanges with clinical OCD experts, young patients appear to be able to suppress their symptoms at school or while with their friends. At home, however, higher levels of significant impairments are reported [21], and symptoms frequently intensify, as patients report that particularly sensitive places, such as their own bedroom or the bathroom, need to be kept clean from external contamination. For this reason, ERP at home is essential, and several studies have highlighted the efficacy of videoconferencing-delivered CBT for OCD [22–27].

In the application of vCBT for pediatric OCD, manual approaches have proven successful. According to Seligman and Ollendick, CBT with ERP involves four phases of treatment: (1) preparation and psychoeducation, (2) hierarchization of compulsions, (3) repeated exposure sessions, and (4) maintenance of treatment success and relapse prevention [28]. In previous studies by our team, we developed a digital treatment manual for pediatric OCD, consisting of these four components and based on the German Therapy Manual for Children and Adolescents [29]. In a 14-week randomized controlled trial (RCT), 60 children and adolescents with OCD were treated with the digital CBT manual, and ERP sessions were performed directly in the patients’ home environment [30]. A significant decrease in symptoms was observed in the treatment group compared with the waitlist control group (Cohen’s *d* = 1.63), showing high patient satisfaction and compliance with the digital treatment approach. A newly developed enhanced CBT package (“eCBT”) containing evidence-based principles of CBT from the Norwegian and Dutch treatment manuals for pediatric OCD, such as psychoeducation, ERP, cognitive interventions, and relapse prevention, achieved a significant improvement in symptoms during a combination of face-to-face sessions, video therapy, and an app system [31, 32].

Until now, however, just a small number of evaluated treatment manuals have been available for pediatric OCD, and despite the given effectiveness of manual-based CBT with ERP approaches, only a few of them have been used in everyday practice. In addition to time factors and expertise, fears that ERP could impair the therapeutic alliance also appear to play a role in some cases [33–35], however, such fears could not be confirmed [36]. Although self-guided interventions alone have been found to be helpful in the treatment of adults or adolescents with compulsions [37–39], these have not been studied much in childhood. Instead, the presence of the therapist or family members, who support the patients as they experience real-life exposure in the ERP sessions and help them face their fears and compulsions, appears to be relevant for the successful implementation of exposures, especially for young children [40]. In many cases, parents and other relatives are affected by the patients’ OCD rituals and suffer from dysfunctional interactions between family members, such as less positive problem solving, high expressed emotions, or less rewarding of independence [41, 42]. Families with children affected by OCD have also reported high levels of parental strain or feelings of distress and guilt, indicating a special need for appropriate support [43]. Therefore, involving parents in ERP sessions can be helpful, as it can lead to a significant improvement in pediatric OCD symptoms and family accommodation [26, 44, 45].

When conducting ERP in the home environments of patients and their families, the therapists’ close supervision of the treatment sessions and real-time guidance of exposures play major roles in facilitating effective therapy. However, the small screen through which the therapist and patient see each other usually leads to limitations in recognizing challenges during ERP. For example, it appears difficult to examine whether the children and adolescents are looking at the anxiety-inducing stimulus during the confrontation and thus being exposed to the stimulus or whether they are averting their gaze and thus avoiding the confrontation. In fact, avoidance or safety behavior during exposures has led to poorer treatment outcomes [46]. Wheaton and colleagues examined the impact of avoidance behavior in OCD patients on treatment outcomes with ERP and found that pretreatment avoidance predicted posttreatment OCD symptom severity [47]. Therefore, providing the therapist with physiological information, such as the patient’s gaze, can add valuable insight. More information on the patient’s engagement with the exposure exercise could be provided by measures of physical tension, which is almost impossible to observe more closely through the screen section. For this reason, the integration of multimodal sensors may be helpful in improving the efficacy of internet-based treatment for OCD [48]. With the help of newly emerging technologies, objective measure of stress can be recorded through heart rate (HR) and heart rate variability (HRV) or electrodermal activity (EDA), gaze data, or movement data [49–52].

### The present study and research questions

In the present study, we aimed to further develop vCBT for children and adolescents with OCD and to supplement it with a multimodal sensor system to conduct a deeper investigation of the physiological and emotional processes that occur during therapy. Thus, we designed a sensor-assisted treatment approach for pediatric OCD called SSTeP KiZ (Smart Sensor Technology in Tele-Psychotherapy for Children and Adolescents with OCD). In a psychotherapeutic trial, 14 vCBT sessions based on a newly revised digital treatment manual were delivered with a focus on exposure exercises in the home environment of the young patients. As primary outcomes, we evaluated the feasibility of the vCBT manual with respect to adherence and therapy content as well as the perceived helpfulness of this approach. Feasibility was also assessed by collecting and evaluating the parents’ barriers to treatment, such as the amount of time that was devoted to therapy, potential conflicts with other activities, the parents’ understanding of the treatment content, or problems at home. We hypothesized good feasibility of our revised online manual and the specific vCBT content, especially in terms of the session content. As secondary outcomes, we investigated the change in OCD symptoms during video-based therapy as well as the effects of the treatment on family accommodation and the general level of functioning. We hypothesized that, at the end of treatment, we would find decreased symptom severity in pediatric OCD, an improved family life, and an increased general level of functioning.

## Methods

### Design

This feasibility study was a pre-post open trial. Data were collected from children and adolescents with OCD and their legal guardians, who provided written informed consent and approval prior to their enrollment. The technical components of the sensor system were developed from the beginning of the funding period until March 2022, with additional adjustments made until the end of the project. The treatment period lasted from April 2022 until the end of March 2023. The Ethics Committee of the Medical Faculty at the University of Tübingen, Germany approved the study (877/2020BO1).

### Participants

Children and adolescents between 12 and 18 years of age with a primary diagnosis of OCD according to the Diagnostic and Statistical Manual of Mental Disorders, Fifth Edition [14] were treated. After the development phase, 20 pediatric OCD patients were included in the treatment with sensor-assisted vCBT, of which the technical components were revised further and made more stable while treating the first six patients, followed by the other 14 patients. On the basis of a pilot study and other face-to-face psychotherapy studies, we planned to include 26 patients in our study, a sample size that was deemed appropriate for meeting the objectives of this study phase [53, 54]. However, due to delays in the development of the complex sensor system, we could include only 20 patients in the end. Inclusion criteria were a Children’s Yale-Brown Obsessive–Compulsive Scale (CY-BOCS) score ≥ 16  [55] and at least one legal guardian and a family home with broadband internet access. Psychiatric medication was allowed during the study, if the dose was stable for 6 weeks prior to the diagnostic session and then continued at the same level during the study. To ensure enough support for the technical equipment and psychotherapeutic exercises during therapy, the children’s living conditions had to be stable. Participants were excluded if they had an IQ below 70, did not speak or understand German, had a psychiatric comorbidity with a higher treatment priority than OCD that made participation clinically inappropriate (e.g., schizophrenia, eating disorder, major depression), or required full inpatient treatment according to the clinical impression. Additional exclusion criteria were lack of motivation, participation in the preliminary study, severe difficulties in handling technical devices, symptoms that were too mild or too severe, or the patient’s preference for onsite treatment. No other psychotherapeutic treatment was allowed while they were participating in the study. If side effects were reported or if the patients wished, they were able to discontinue treatment at any time, and we assisted them in finding another treatment option. Table 1 presents the patients’ demographic characteristics.

**Table 1:** Patients’ demographic characteristics (*N* = 20)

Age	
*Mean* (*SD*)	15.7 (1.78)
*Range*	12–18
Gender (% male)	45
Country of origin (% parents)	
*Germany*	92.5
*Another European country*	5
*Non-European country*	2.5
Marital status	
*Single/separated/divorced/widowed*	5
*Married/living together*	15
Education (% parents)	
*University or equivalent*	35
Previous treatment	
*Psychiatric*	6
*Psychotherapeutic* (*ambulatory setting*)	8
*Psychotherapeutic* (*inpatient treatment*)	2
*Experience with Exposure and Response Prevention* (*ERP*)	3
*Pharmacological*	3
One or more comorbid diagnoses	15
*Anxiety disorders*	9
*PTSD/Adjustment disorder*	4
*ADHD*	2
*Depression*	1
*Other emotional disorders*	3

All of the *N* = 20 patients included in the study completed all 14 sessions of videoconferencing-delivered CBT

### Technical devices

In SSTeP KiZ, experts from different fields, such as psychotherapy, psychiatry, IT and software development, graphic design, and health economics, developed a multi-modal sensor system for pediatric OCD. In therapy sessions, we recorded stress with the ECG, fixation patterns with the eye tracker, and movement-induced changes in heartrate with movement sensors. An initial analysis of five OCD patients from SSTeP KiZ showed that it was possible to identify stress and repetitive compulsive behavior by utilizing these various sensor modalities [56]. In this pilot study, the data were used offline to record and investigate the physiological and emotional processes in patients with OCD during ERP.

To record the physiological data, the patients received a tablet from us as an aggregator device along with the sensors, which they applied and connected themselves before every therapy session. The therapists accompanied the patients during the exposures so that the therapists could instruct patients precisely and help them connect the sensors to the tablet. The technical system components have already been described in detail [57]. Here, the data were prepared to be streamed to the therapist and saved in predefined data formats with synchronized timestamps for post hoc analysis. The sensor data were processed on the aggregator tablet and then synchronized to a secure cloud platform for offline data analysis (meerfarbig GmbH/Frankfurt Main). On a second tablet, the program for the video sessions and the University Hospital Tübingen’s cloud were installed, where the therapy materials from the individual sessions were saved. The sensor data transmitted by the patient and the completed questionnaires were processed in a web application for the therapist. The web application for the patients, where they evaluated the progress and success of their therapy, consisted of a standard questionnaire interface in which an item was presented with one of the common response formats (Likert scale, multiple choice, and free text). To motivate patients to consistently complete the questionnaires, a gamification approach was integrated. The data were stored on the internal platform of the University Hospital Tübingen IMeRa (Integrated Mobile Health Research Platform).

### Procedure

**Fig. 1:**
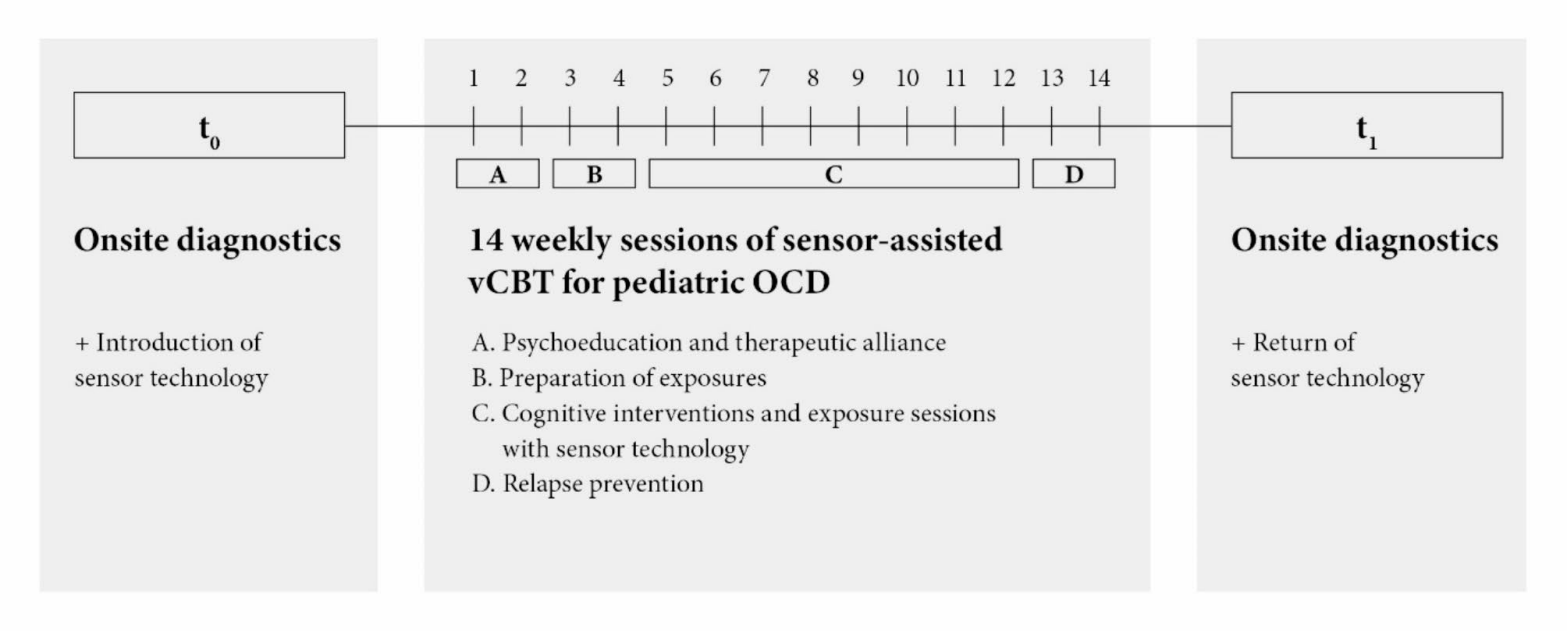
Study process. After an onsite diagnostic assessment (t0) and technical introduction, patients received 14 manual-based CBT sessions via videoconferencing. After completing the treatment, patients underwent another diagnostic assessment at the Child and Adolescent Psychiatry Tübingen (t1)

Participants were recruited from local psychiatrists, psychologists, the German OCD Society, and our advertising campaign on Google AdWords that was linked to our study website. We conducted the study in collaboration with our IT department and discussed data security issues with the IT and data security specialists at our clinic. After participants contacted the study investigators, the families were invited to attend an online video conference to learn more about the general eligibility requirements. If attendees met the preliminary inclusion criteria, a baseline preassessment (t0) was conducted during an in-person appointment at the hospital with an independent diagnostician who was not involved in the treatment process. The results were then reviewed and discussed by the study team of the Child and Adolescent Psychiatry Tübingen. We used our hospital’s medical history form to gather demographic information, treatment history, and family history from patients and parents. Additionally, we gave patients an intelligence exam (Culture Fair Test 20-R [58]) to evaluate their basic cognitive abilities and general fluid intelligence. The final decision on participation was then made. After the diagnostic evaluation, the diagnostician gave the patients and their families a detailed introduction on how to use the sensor system. The patients performed various tasks with the sensor system (e.g., walking up and down stairs, sorting things) and in the process became familiar with the operation of the individual sensors. Subsequently, the main study began with the enrollment of the 20 patients who each received 14 weekly video therapy sessions of approximately 90 min each. Family members could be directly involved in the therapy and were given advice on how to handle the symptoms at home. After the study was completed, an additional diagnostic assessment (t1) with the independent diagnostician was carried out on site at the Child and Adolescent Psychiatry Tübingen, and the results were discussed with the study team. For a detailed overview, the study process is illustrated in Fig. 1.

**Fig. 2:**
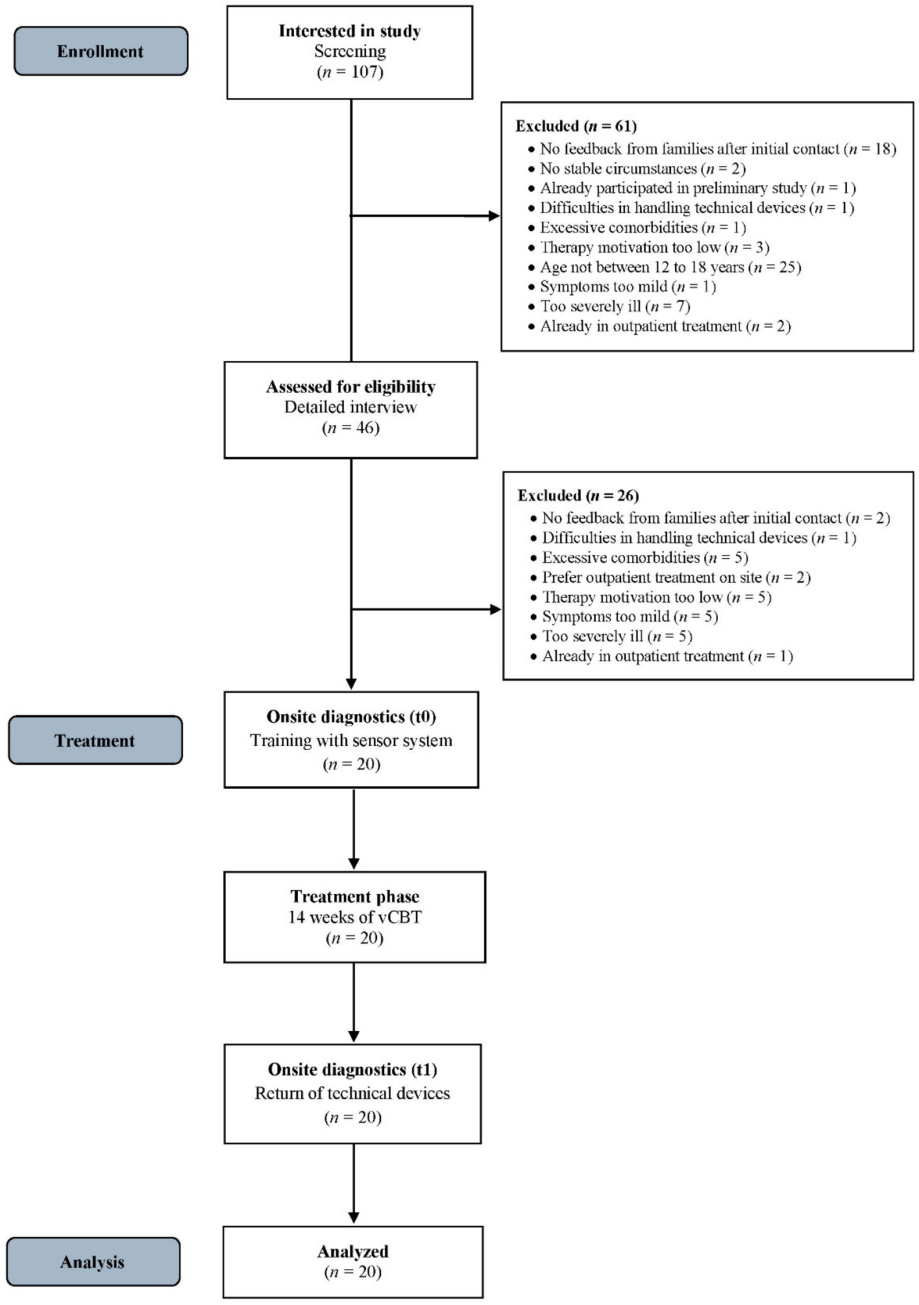
Participant flow diagram. This figure shows the participant flow diagram. *N* = Number of participants

The psychotherapeutic treatments were carried out by two licensed psychotherapists, each of whom treated 10 patients. Both therapists were trained in CBT for children and adolescents and already had experience with internet-based psychotherapy for OCD from previous clinical trials. The data were recorded pseudonymously and transmitted in encrypted form, and the research team at the University Hospital Tübingen evaluated the data. The data will be saved for 10 years and will be deleted at the participants’ request. Figure 2 presents an overview of the study flow.

### Manual

The SSTeP KiZ manual incorporated evidence-based principles of CBT from the German Reference Manual for pediatric OCD [29], adapted to the online-based intervention. Each patient received the standardized treatment in accordance with this manual. It consisted of four modules with every session containing a briefing on the objectives, a progress assessment of previous and ongoing exercises, the presentation and practicing of new material, and a discussion of the following week’s homework. The manual also included psychoeducation stories, instructions on how to carry out self-management exposures in the home environment, or a case study of a former patient dealing with OCD symptoms. Furthermore, videos were recorded by our department to train patients how to apply the sensor system correctly at home.

*(1) Sessions 1–2: Establishing the therapeutic relationship and psychoeducation*.

In this phase, patients and therapists got to know each other via the video sessions. The therapist provided online material in a secure cloud so that the patient and their parents could prepare for the therapy session. After the session, any new psychoeducational materials that had been discussed were uploaded back to the cloud so that families could access them. In addition, an individual bio-psycho-social stress model was developed together with the patients and their parents.

*(2) Sessions 3–4: Hierarchization of compulsions and preparation of exposures*.

In this phase, all existing OCD symptoms were recorded in detail and mapped into a hierarchy of 1-100 in order to precisely define the severity of the individual symptoms. Along with the cognitive interventions, the OCD hierarchy provided the basis for the subsequent exposure sessions. Strategies were discussed with the parents on how to best support the children at home and during the exposure exercises. In Session 4, a first practice session was held to familiarize the child with the procedure that would be applied during the ERP sessions.

*(3) Sessions 5–12: cognitive interventions and implementation of ERP*.

These sessions were the key element of vCBT for OCD. In the therapy sessions, exercises from the obsessive-compulsive hierarchy were chosen and practiced with increasing levels of difficulty together with the therapist directly in the home environment, where most of the symptoms usually occurred. In addition to the exposure exercises, patients were given additional cognitive interventions to improve how they handled obsessive thoughts and actions. Patients and their families were also encouraged to carry out self-management exercises between therapy sessions. The study team offered the children and their families technical support if they had difficulties applying the sensor system.

*(4) Sessions 13–14: final exercises*,* relapse prevention*,* and maintenance of treatment success*.

At the end of the treatment, final exercises could be carried out in Session 13. The focus during these two sessions was on relapse prevention to maintain the success of the treatment in the long term and to discuss additional strategies that the children and their families could implement at home.

### Primary outcome measures

#### Measures of manual adherence, therapy content, and feasibility

The Manual Rating Form is a checklist with five items that the therapist used after each individual therapy session to record the extent to which the content of the session could be carried out in accordance with the manual or whether content was missing in the individual session or was too much for the session. The answers were given on a 3-point Likert scale ranging from 1 = *Not at all* to 3 = *Very much*.

A session checklist was developed to monitor each sensor-based vCBT treatment session and to record deviations from the SSTeP KiZ treatment manual. The checklist was completed by the therapist after each session.

The Summary Therapist Feedback Form was used to assess the ease of manual implementation [59]. Questions such as “How easy was it to understand the content of the manual?”; “How easy was it to conduct the treatment as outlined by the manual?”; “Did you feel 14 sessions were sufficient to accomplish all of the treatment goals?” were answered on a 7-point Likert scale ranging from 1 = *Not at all* to 7 = *Very much*.

General feasibility was measured as treatment drop-out before completion of the planned 14 vCBT sessions.

In addition, we designed implementation and satisfaction questionnaires for the patients, the parents, and the therapists at t1 to assess the suitability of the therapy content, the therapy motivation, and the quality of family life. The answers were given either on a 4-point Likert scale ranging from 1 to 4 (1 = *I agree*, 2 = *I somewhat agree*, 3 = *I somewhat disagree*, and 4 = *I disagree*) or in free-text fields.

To evaluate the perceived barriers to treatment, we used the self-report questionnaire Barriers to Treatment Participation Scale (BTPS) [60]. The BTPS consisted of 46 items that were answered on a 5-point Likert scale (1 = *was never a problem* to 5 = *was very often a problem*). It assessed potential problems with the time devoted to therapy, the therapist, participation in the treatment sessions, or difficulties in the families’ social environments.

### Secondary outcome measures

#### Clinical measures

The Children’s Yale-Brown Obsessive-Compulsive Scale (CY-BOCS) as the gold standard in assessing the severity of OCD symptoms is a clinician-rated, semi-structured interview for measuring treatment response [55]. It consisted of 10 items assessing five dimensions (time occupied by symptoms, distress, interference, resistance, and degree of control over symptoms) of obsessions and compulsions, based on interviews with each child and a parent. The CY-BOCS total score ranges from 0 to 40 (clinical cut-off = 16). According to the suggestions offered by Babiano-Espinosa and colleagues, remission is defined as a CY-BOCS total score ≤ 12 and treatment response as ≥ 35% symptom reduction on the CY-BOCS and a CGI-I score of 1 or 2 [31].

The Clinical Global Impression (CGI) is used to measure symptom severity, treatment response, and efficacy in treatment studies. Its scales measure severity and improvement. The Severity Scale (CGI-S) is strongly correlated with the CY-BOCS total score in pediatric OCD patients and is a 7-point scale ranging from 1 = *Normal* to 7 = *Among the most extremely ill patients* [61]. The Improvement Scale (CGI-I) was used to assess overall clinical improvement based on observed symptoms and reported impairment [61]. It is also a 7-point scale ranging from 1 = *Very much improved* to 7 = *Very much worse.*

The Children’s Global Assessment Scale (CGAS) was used to examine patients’ overall level of general functioning [62]. The clinician assessed a range of aspects of psychological and social functioning and gave the child or young person a single score between 1 and 100 based on their lowest level of functioning. The score assigned them to one of 10 categories that ranged from 1 to 10 = *Needs constant supervision* to 91–100 = *Superior functioning*.

The Child Behavior Checklist (CBCL/6-18R) is a parent-report form designed to assess a wide range of child behavioral and emotional problems [63]. The counterpart for adolescents is the Youth Self-Report (YSR/11-18R), which is a self-report questionnaire for children and adolescents used to evaluate various behavioral and emotional problems [64].

### Data analysis

All statistical analyses were performed in R 4.2 [65] and with IBM SPSS Statistics (Version 28). Measurements of feasibility, acceptance, and implementation were evaluated descriptively with sample means, standard deviations, and ranges. The clinical measures were assessed with *t* tests, comparing the total scores from t0 and t1. We adjusted the *t* test analyses for multiple testing by applying the Benjamini-Hochberg correction method, which controls the false discovery rate, i.e., the expected proportion of false discoveries amongst the rejected hypotheses [66]. Cohen’s *d* was used as an effect size measure with a small effect beginning at 0.2, a medium effect beginning at 0.5, and a strong effect beginning at 0.8.

## Results

In the following, the results for manual adherence, perceived helpfulness of the therapy content, and the feasibility of the entire treatment process are presented from the perspectives of the patients, their parents, and the therapists. In addition, the secondary outcomes from the clinical data are presented.

### Primary outcomes

#### Measures of manual adherence, therapy content, and motivation by therapists

**Fig. 3:**
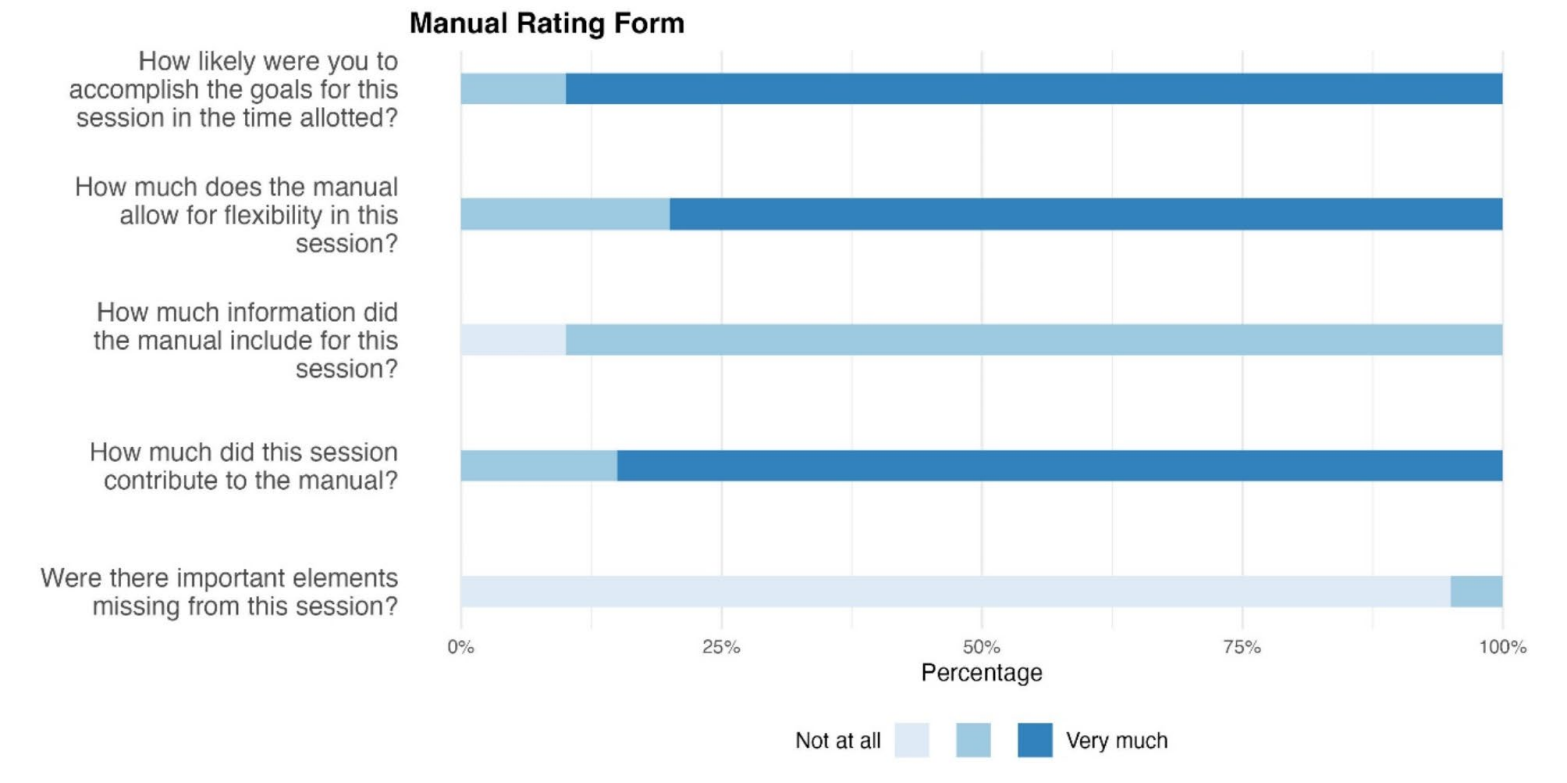
Manual Rating Form. In this figure, the average results for the Manual Rating Form after each session are shown in percentages

(1) Manual Rating Form. In Step 1, we calculated the means of the five items for each of the four treatment modules. Regarding manual adherence, there were no significant differences between the four modules over time, indicating that the manual could be used in all treatment periods. In Step 2, the distributions of answers for each of the five different items were calculated across the 14 sessions as presented in Fig. 3.

According to the Manual Rating Form, the feasibility and practicability of the entire manual for online-based CBT was rated very high. Therapists stated that they achieved their goals for the individual sessions in the time available. They rated the level of flexibility during the sessions as sufficient and the amount of information as adequate.

*(2) Summary Therapist Feedback Form*.

**Fig. 4:**
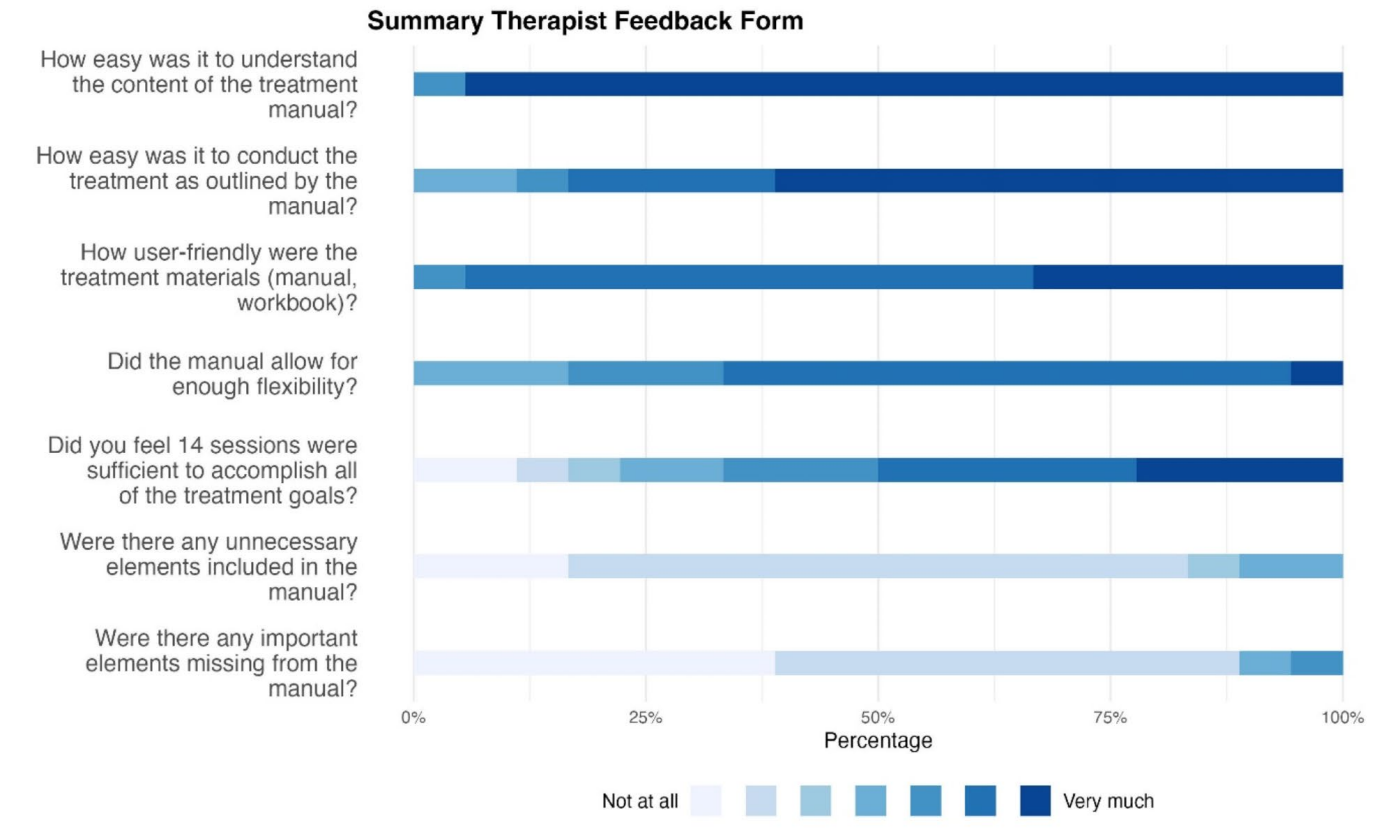
Summary Therapist Feedback Form. The results of the Summary Therapist Feedback Form are shown as percentages to measure the general feasibility of the manual during the 14 vCBT sessions

The final post-treatment questionnaire (Summary Therapist Feedback Form) showed a high level of user-friendliness, easy comprehensibility of the manual’s content, and a high degree of general feasibility. The therapists rated the number of sessions and the content of the individual sessions as sufficient.

*(3) Manual Implementation Questionnaire*.

**Fig. 5:**
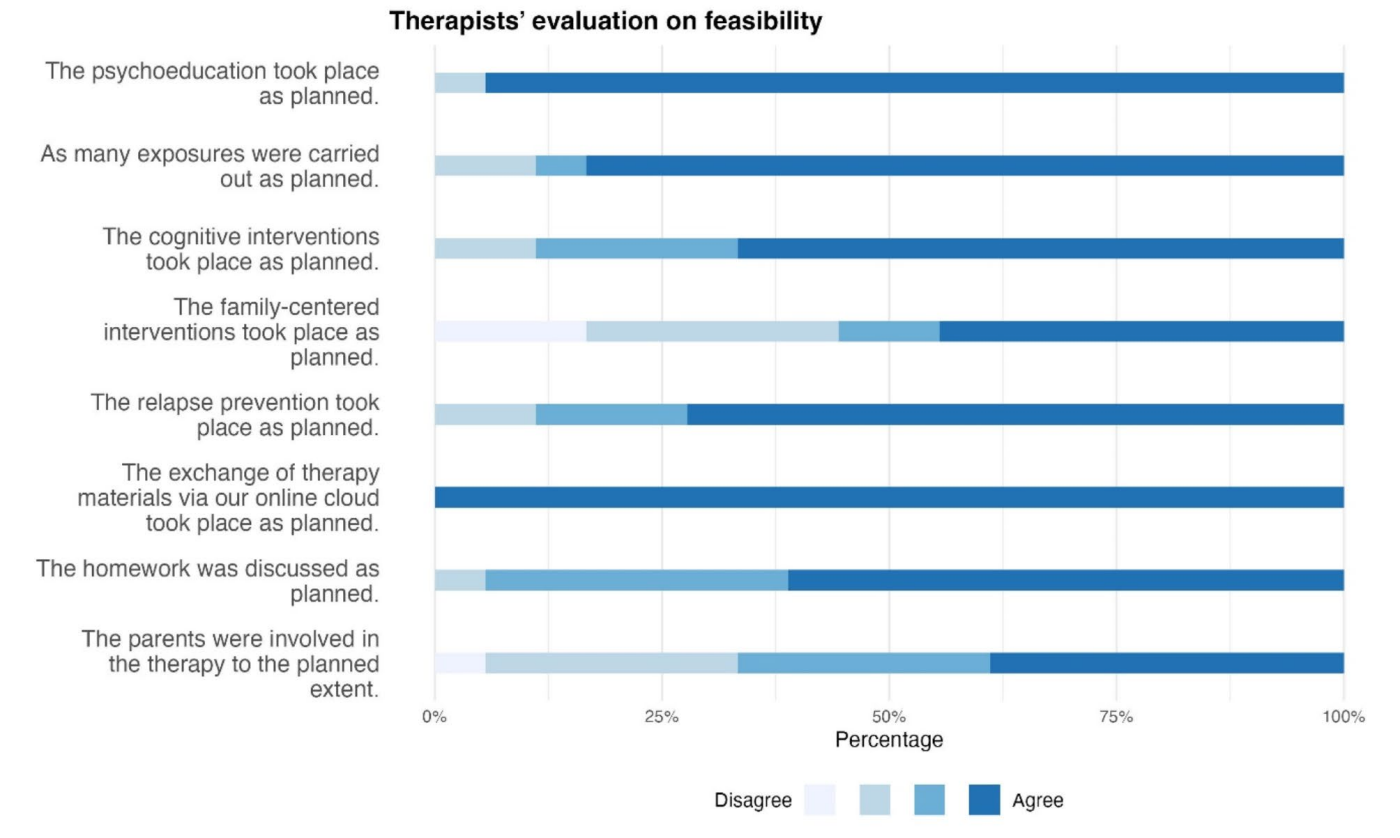
Implementation questionnaire. The figure shows the results from the Implementation Questionnaire, which was designed to assess all specific content during treatment

Based on the Implementation Questionnaire by therapists, the exchange of therapy materials via the cloud and the discussion of homework were carried out as planned, as well as the implementation of therapy content, with some limitations for the family-centered interventions and parent involvement.

*(4) Therapists’ Final Questionnaire*.

**Fig. 6:**
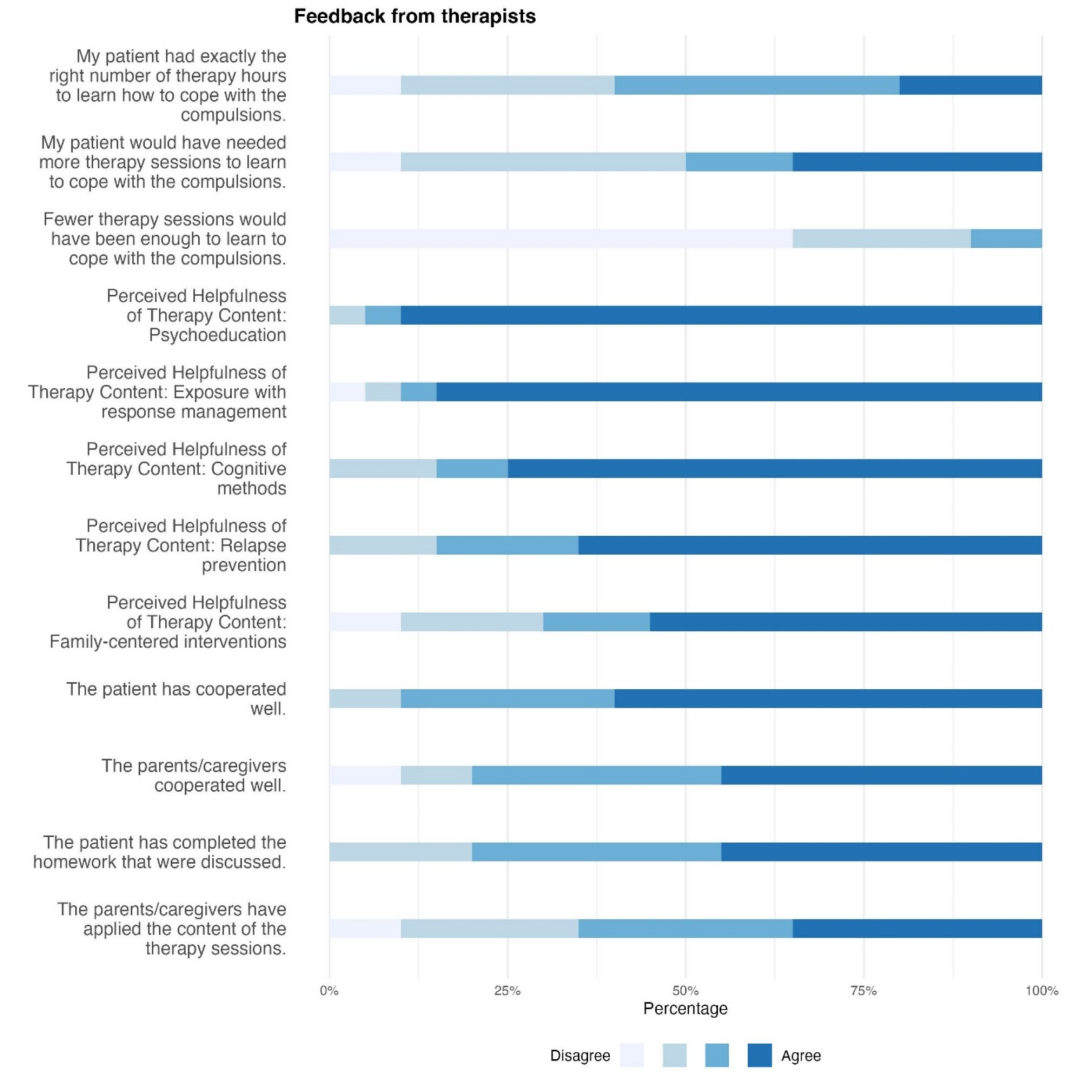
Therapists' Final Questionnaire. The figure presents answers about the number of therapeutic sessions, perceived helpfulness of the therapy content, and patient’s motivation for online-based treatment as percentages

Therapists rated all therapy content as helpful, with different gradations. Patients’ motivation was rated high in terms of cooperation and homework processing. Parents’ motivation was rated high to moderate, and the content was less well implemented by some parents.

Measures of Feasibility and Therapy Content by Patients and Parents.

**Fig. 7:**
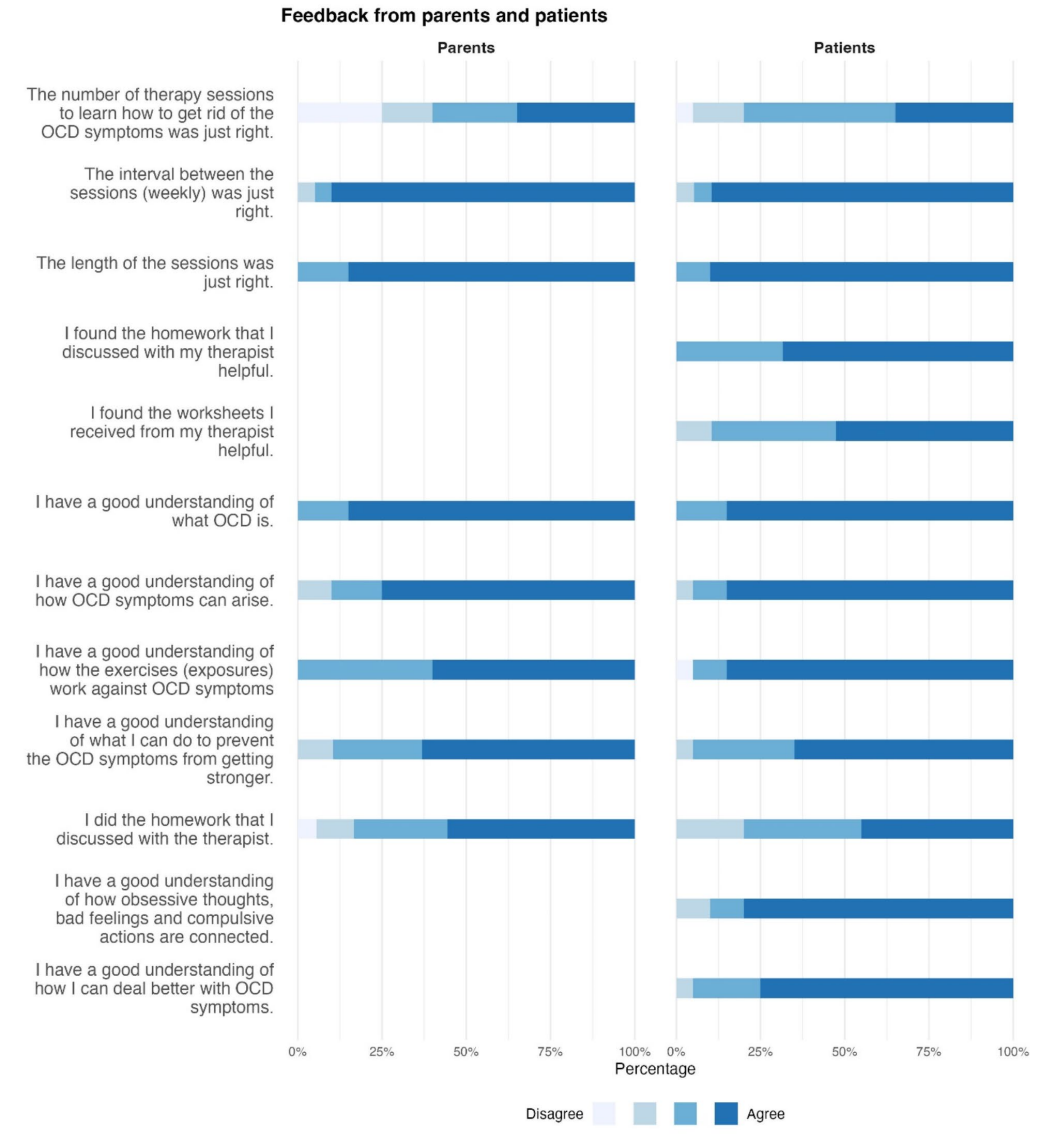
Patients' and parents' final questionnaire. The results from the Final Questionnaires from the patients and their parents are displayed as percentages. 20 patients and parents answered the questions, with the exception of one parent couple who did not respond to the two items “I felt that I was sufficiently involved in the therapy” and “The amount of parent involvement was exactly right”

(1) Patients’ and Parents’ Final Questionnaire. The patients and parents completed the Final Questionnaire on the number of therapy sessions and content (Fig. 7).

Some patients stated that they needed more sessions to get rid of the OCD symptoms. Satisfaction with the time between the sessions and the session length was rated high. Therapy content was perceived as very helpful by patients and parents. Patients stated that homework and worksheets were helpful and that they gained a better understanding of OCD and how to deal with this disorder.

(2) Barriers to Treatment Participation Scale. Concerning the perceived barriers to treatment, parents reported low barriers and did not consider the treatment too demanding or not helpful. On the contrary, parents rated the treatment as suitable for their and their children’s needs. The parents rated the interaction with the therapist as satisfying and indicated that it was never a problem to understand the therapy content.

### Secondary outcomes

#### (1) Clinical measures

We compared the values measured at t0 and t1 using *t* tests (Table 2). We calculated the percentages of patients who were in remission after treatment and those who responded to treatment.

**Table 2:** Pre-post comparison of treatment effects

Instrument	Pre		Post		t test		
	*M*	*SD*	*M*	*SD*	*t*(*19*)	p*	Cohen’s *d* (95% CI)
CY-BOCS	24.35	3.33	11.30	7.05	8.37	< 0.001	1.87 (1.13–2.60)
CGI-S	4.95	0.95	2.05	1.43	9.72	< 0.001	2.17 (1.35–2.98)
CGAS	45.00	9.49	77.80	15.13	8.78	< 0.001	1.96 (1.20–2.71)
CBCL	59.95	7.79	54.65	6.93	3.30	0.008	0.74 (0.24–1.23)
Internal	63.60	8.56	56.05	12.88	2.36	0.053	0.53 (0.05–0.99)
External	51.45	6.88	45.05	12.61	2.08	0.076	0.47 (0.002–0.92)
YSR	59.80	8.53	57.15	7.29	1.69	0.139	0.38 (0.08–0.83)
Internal	63.00	9.67	60.35	8.45	1.55	0.154	0.35 (0.11–0.79)
External	51.20	8.34	50.35	7.73	0.74	0.467	0.17 (0.28–0.61)

* The Benjamini-Hochberg correction was applied to adjust for multiple testing

The *t* tests between t0 and t1 revealed a significant decrease in OCD symptom severity, measured with the CY-BOCS. Overall, the CY-BOCS total score decreased on average by 53.82% (13.1 points). Remission was achieved in 12 of 20 patients, and 15 of 20 patients were considered treatment responders.

The clinician-rated symptom severity (CGI-S) also revealed a significant reduction from pre-treatment to post-treatment. A positive change in the symptomatology measured with the CGI-I could be observed in most of the patients, as 17 of 20 participants (85%) were rated *much improved* or *very much improved* (*M* = 1.65, *SD* = 0.96).

Improvement in the level of social functioning (CGAS) from pre-treatment to post-treatment was also significant.

The child behavior checklist (CBCL) parent-report showed significant improvement in the behavioral and emotional problems of the children between pre-treatment and post-treatment. The results from the internal and external parent ratings were not significant. The analysis of the youth self-report (YSR) revealed no significant effects.

### (2) Family Life

In the final assessment, 85% of patients and their parents *agreed* or *rather agreed* that their family life had improved since vCBT.

## Discussion

In the present study, our primary goal was to examine treatment feasibility and manual adherence for a newly developed vCBT approach for children and adolescents with OCD. As secondary outcomes, we additionally investigated the course of clinical symptoms between the pre- and post-treatment assessments. The primary results of our study suggest that vCBT with a multimodal sensor system, used for the first time in the treatment of pediatric OCD, is sufficiently applicable and, in conjunction with the newly revised digital treatment manual, supports the success of the therapy. A total of 107 patients applied to participate in our study, 20 of whom were included, reflecting a high demand for OCD treatment in children and adolescents.

The evaluation of the vCBT manual that was adapted for online use showed that the therapists were able to achieve the goals of the individual sessions in the given time per week. The therapists rated the adaptability of the content of the manual to the individual sessions as very good and the amount of information for each session as exactly right. The extent to which the individual sessions contributed to the implementation of the manual was rated as great, and the therapists stated that no important content was missing from the sessions. In the final questionnaires, the therapists summarized that the content of the manual was easy to understand and that they could easily carry out the treatment in accordance with the manual.

These observations are consistent with findings by Lisi et al. [23], who studied *n* = 144 adult patients with moderate to severe OCD in a two-group comparison of manualized CBT. They showed that both face-to-face and videoconferencing-delivered group-based CBT achieved significant, reliable, and statistically equivalent improvements in OCD symptoms, suggesting that clinician-led vCBT may be a helpful treatment approach. In the present study, all content was perceived as feasible and helpful, with some limitations to parent involvement. The homework was discussed as planned, and the families were also included in the treatment in accordance with the manual. The number of sessions was perceived as sufficient, and the patients’ motivation was rated as high in terms of cooperation and homework processing. Parents’ motivation was classified as high to moderate; however, some parents did not cooperate as much and did not apply the content of the sessions themselves.

The evaluations of the patients and their parents in the final questionnaires showed that the number of sessions was perceived as sufficient for handling the OCD symptoms, although some families would have wanted more therapy sessions. The patients and parents were highly satisfied with the lengths of the sessions and the time between the sessions, and both rated the treatment content as very helpful. Patients and parents indicated that they understood how ERP works against OCD symptoms and what they could do to prevent relapse. None of the patients dropped out of SSTeP KiZ, and all families completed the treatment, indicating that this manual-based approach was feasible for young patients with OCD and their parents. The assessment of the perceived barriers to treatment participation also revealed good feasibility of the treatment, as parents did not consider the treatment to be too demanding, a finding that is comparable to Weidle et al.’s [24] observations. On the contrary, parents rated the treatment as helpful and suitable for their and their children’s needs. Some parents stated that they experienced stress in their lives along with the treatment and reported a serious illness or the death of a loved one during the time when they participated.

As secondary outcomes, the clinical measures were investigated with respect to improvement in symptoms and quality of life. The comparison between pre- and post-treatment revealed a significant decrease in the severity of OCD symptoms, reflecting a large effect, which is similar to other studies in which psychotherapy was delivered via videoconferencing [22, 27]. Overall, the CY-BOCS total score decreased on average by 53.82% (13.1 points), while a positive change in the symptomatology could be observed in most patients, as 17 of 20 participants (85%) were rated *much improved* or *very much improved*. Patients’ overall level of general functioning also improved significantly. A total of 15 of 20 patients were considered to be treatment responders, and remission was achieved in 12 of these 20 patients. The results are comparable to other online-based approaches for pediatric OCD [25, 31, 54, 67].

Patients and parents reported an improvement in family life after 14 weeks of treatment. For two patients, the online treatment hardly made any difference to the clinical values, which suggests that not all patients benefit equally from online treatment. Despite the evidence of the success of vCBT, research has highlighted that the efficacy of online-based treatment depends on various factors, such as the severity of the disorder, specific comorbid conditions, or OCD-related avoidance behavior [68–71]. In our case, both patients were severely ill at the beginning of treatment, and both suffered from comorbid diagnoses (social phobia, generalized anxiety disorder, attention deficit hyperactivity disorder). A manual with a transdiagnostic approach could be beneficial here, in which not only the treatment of a single disorder is targeted but also the comorbid diagnoses [72].

The core element of SSTeP KiZ was the implementation of manual-based vCBT in the home environment with a focus on ERP, which was experienced by parents, patients, and therapists as enormously helpful, also reflected by the significant reduction in severe OCD symptoms in some cases. Similar results were observed in a pilot study with *n* = 15 adults with OCD, where patients received 16–18 twice-weekly, 90 min sessions of videoconferencing-delivered ERP, which were associated with a significant improvement in OCD symptoms [73]. In a large cohort of 3,552 adults with OCD, videoconferencing-delivered ERP significantly reduced obsessive-compulsive and comorbid symptoms and improved quality of life [22]. For early-onset OCD, an RCT with videoconferencing-delivered, family-based CBT including *n* = 22 children between 4 and 8 years of age also showed a significant reduction in obsessive-compulsive symptoms and a high level of acceptance with the online-based approach [26]. In digital interventions, exposure exercises can be carried out directly in the situations where most of the OCD symptoms are triggered, accompanied by relatives, and generalized to other scenarios in patients’ daily lives. vCBT works across geographical distances and makes it possible for the therapist to be present in the family’s home environment at different times whenever symptoms occur. Moreover, online treatment appears to be a great relief for parents, as they do not have to organize additional travel, and siblings can be supervised more easily in the home environment, which lowers the barriers to treatment participation for families [11, 74].

In summary, the newly revised SSTeP KiZ manual can be considered highly usable by therapists, patients, and parents and represents a significant contribution to the further development of vCBT in general practice. The composition of the manual, which consists of the four core elements, seems to be very helpful for patients and their families and thus represents a promising approach for vCBT in pediatric OCD. In many cases, digital treatment manuals tend to be a simple transfer of paper and pencil content into an online format to ensure an evidence-based approach and the same systematic conditions for all participants. However, this simple transfer interferes with the desire of young people for self-management, personalization, and control [75, 76], thus producing a gap between the existing needs of young patients and the issues typically addressed by digital treatment tools from clinical research [77, 78].

By contrast, developing a digital manual in the sense of a participatory design approach that really appeals to children and adolescents in terms of graphics and content means involving them in the creative process from the very beginning [79]. Therefore, technology-based innovations should be perceived as services for end users, rather than just therapeutic products (Mohr et al., 2013). In fact, user-centered design approaches can significantly increase young people’s intrinsic motivation to engage with technical devices (Chatterjee et al., 2022).

Meanwhile, some manual-based treatments for children and adolescents have also been implemented in the form of an app with gamification. In SSTeP KiZ, we developed a game-based incentive for the patient web application, which was used to complete the daily and weekly questionnaires. We received feedback from potential users on the design, user experience, and structure of the gamification modules at different points during treatment. The young people responded positively to the illustrations and the overall interface design. Another promising approach to facilitate treatment for various mental disorders in children and adolescents is serious gaming. A manualized, parent-assisted serious game called “Zirkus Empathico” was applied in a multicenter RCT to strengthen socio-emotional skills, such as empathy and emotion recognition in children with autism [80]. Moreover, it might also be possible to further extend the already existing gamification approaches in online-based manuals to support patients in carrying out self-management exercises at home without a therapist. According to this idea, a therapist avatar or the integration of a chatbot that could independently interact with the patient might be suitable for further enhancing online-based interventions. Multimodal sensor technology seems to be helpful for gaining additional information on patients’ physiological and emotional states. In research on children with autism spectrum conditions, a humanoid robot was successfully used to detect the child’s emotions and arousal with different sensors [81]. An initial evaluation of the transfer of physiological data has already been carried out, revealing a potential automated identification of stress and repetitive compulsive behavior by applying the sensor system during exposures [56]. Patients’ self-assessments and external assessments are to be analyzed in a subsequent paper, investigating the connection between physiological measures and diagnostic evaluations.

### Limitations

The evaluation of the final questionnaires showed that the manual was easy to implement, although only two therapists assessed its feasibility. In subsequent studies, to improve generalization, a larger group of therapists should be asked about the feasibility of the manual. As our study was a single-armed feasibility trial without a control group, only limited conclusions could be drawn about the efficacy. The stability of the effects could not be evaluated, as no follow-up assessment was conducted in this study. Due to the single-arm study design, the complete blinding of the participants and the diagnostician was not possible. An independent rater carried out the diagnostic assessment and reported the results in a range, after which the data were discussed by the research team. Thus, the diagnostic examination was not completely independent, but it is unlikely that the data were falsified because the predefined range was maintained. In follow-up studies, a completely independent rater should carry out the diagnostic assessment. During the treatment period, there were limitations in data transmission during the sessions so that the evaluation of the sensor data was mainly carried out offline. In future studies, data streaming should be made more robust to transmit the physiological processes to the therapist in real time. As this study was a technically pioneering and innovative approach, this form of treatment might not be suitable for all children and adolescents with OCD. At the same time, there were many exclusion criteria that would apply to any videoconferencing-delivered CBT study, such as low motivation, a preference for a face-to-face approach, or a level of illness that is too severe or too mild. Similar to other videoconferencing-delivered feasibility studies, the results cannot be easily generalized to all pediatric patients in routine care and should be investigated in further studies. Although this treatment was primarily delivered online, thereby facilitating participation for the families, participants still had to come to our hospital for the diagnostic assessments and the technical introduction. In the future, we are considering mailing the sensors and carrying out the diagnostic assessments online to prevent the need for an onsite visit. Future trials with a larger sample and control groups are needed to investigate the efficacy of such a technology-based approach with young people suffering from OCD.

## Conclusions

The promising results of this feasibility study indicate a successful application of manual-based vCBT in pediatric OCD supported by a sensor system. The newly revised manual proved to be easy to implement as part of the 14-week online treatment. Patients, parents, and therapists were satisfied with this approach and rated the treatment as helpful. A significant reduction in OCD symptoms was observed in the vast majority of patients. Although additional efficacy studies in a broad sample of OCD patients should follow, the results highlight the importance of implementing manual-based vCBT with ERP directly in the patients’ home environment, where OCD symptoms primarily occur. In this sense, the involvement of family members was also found to be effective by patients and their parents in helping them manage symptoms at home, with no major barriers to participation in the treatment reported. During this pilot study, sensor data from exposure sessions could be used offline, whereby initial evaluations showed a potential automated identification of stress and repetitive compulsive behavior. Future studies with OCD patients are needed to investigate the underlying physiological and emotional processes during ERP and to transmit data in real time to the therapist for the better adaptation of sessions to the individual patient.

## Data Availability

The datasets used and analyzed during the current study are available from the corresponding author, KH, on reasonable request. The data are not publicly available to protect participants’ privacy.

## Abbreviations

BTPS: Barriers to treatment participation scale
CBCL: Child behavior checklist
CBT: Cognitive behavioral therapy
CGAS: Children’s global assessment scale
CGI-I: Clinical global impression– improvement
CGI-S: Clinical global impression– severity
CY-BOCS: Children’s yale-brown obsessive–compulsive scale
EDA: Electrodermal activity
ERP: Exposure with response prevention
HR: Heart rate
HRV: Heart rate variability
IMeRa: Integrated mobile health research platform
OCD: Obsessive-compulsive disorder
RCT: Randomized controlled trial
SSTeP KiZ: Smart sensor technology in tele-psychotherapy for children and adolescents with OCD
vCBT: Cognitive behavioral therapy delivered via videoconferencing
YSR: Youth self-report
